# Trans, Trans-Farnesol Enhances the Anti-Bacterial and Anti-Biofilm Effect of Arachidonic Acid on the Cariogenic Bacteria *Streptococcus mutans* and *Streptococcus sobrinus*

**DOI:** 10.3390/ijms252111770

**Published:** 2024-11-01

**Authors:** Farah Haj-Yahya, Doron Steinberg, Ronit Vogt Sionov

**Affiliations:** Institute of Biomedical and Oral Research (IBOR), Faculty of Dental Medicine, The Hebrew University of Jerusalem, Ein Kerem Campus, Jerusalem 9112102, Israel; farah.hajyahya2@mail.huji.ac.il (F.H.-Y.); dorons@ekmd.huji.ac.il (D.S.)

**Keywords:** anti-bacterial, biofilm, arachidonic acid, dental caries, *Streptococcus mutans*, trans, trans-farnesol

## Abstract

Background: *Streptococcus mutans* and *Streptococcus sobrinus* are Gram-positive bacteria involved in the development of dental caries, as they are able to form biofilms on tooth enamel, ferment sugars into acids, and survive under acidic conditions. This ultimately leads to a local lowering of the pH value on the tooth surface, which causes enamel cavities. Hypothesis: One measure to reduce caries is to limit the growth of cariogenic bacteria by using two anti-bacterial agents with different mechanisms of action. The hypothesis of this study was that the anti-bacterial activity of ω-6 polyunsaturated arachidonic acid (AA) against *S. mutans* and *S. sobrinus* can be enhanced by the sesquiterpene alcohol trans, trans-farnesol (t,t-farnesol). Methods: The anti-bacterial activity of single and combined treatment was determined by the checkerboard assay. Bacterial viability was assessed by live/dead SYTO 9/propidium iodide (PI) staining on flow cytometry. Anti-biofilm activity was determined by MTT metabolic assay, crystal violet staining of biofilm biomass, SYTO 9/PI staining by spinning disk confocal microscopy (SDCM) and high-resolution scanning electron microscopy (HR-SEM). Results: t,t-Farnesol lowered the minimum inhibitory concentration (MIC) and the minimum biofilm inhibitory concentration (MBIC) of AA at sub-MICs. AA reduced the metabolic activity of preformed mature biofilms, while t,t-farnesol had no significant effect. The enhanced anti-bacterial effect of the combined t,t-farnesol/AA treatment was further evidenced by increased PI uptake, indicating membrane perforation. The enhanced anti-biofilm effect was further verified by SDCM and HR-SEM. Gene expression studies showed reduced expression of some biofilm-related genes. Conclusions: Altogether, our study suggests a potential use of the two naturally occurring compounds arachidonic acid and t,t-farnesol for preventing biofilm formation by the cariogenic bacteria *S. mutans* and *S. sobrinus*. These findings have implications for caries prevention.

## 1. Introduction

*Streptococcus mutans* and *Streptococcus sobrinus* are facultative anaerobic Gram-positive bacteria naturally inhabiting the oral cavity [1]. They are among the main etiological factors of dental caries and usually act together with other microorganisms such as the fungus *Candida albicans* [1,2,3,4]. *S. mutans* and *S. sobrinus* possess multiple pathogenic and cariogenic mechanisms. Through the mechanism of adhesion to a solid surface, the cariogenic bacteria are able to colonize the tooth enamel and form bacterial biofilms, so-called dental plaques [1,2,4]. Together with their ability to produce organic acids by fermentation of carbohydrates (acidogenicity) and survive under acidic conditions (aciduricity), the presence of *S. mutans* and *S. sobrinus* in dental plaques leads to a localized decline in pH at the tooth surface [1,4]. The acidification leads to demineralization of calcium and phosphate present in the hydroxyapatite crystals of tooth enamel, resulting in the formation of cavities known as dental caries [5,6]. The three-dimensional biofilm structure of the dental plaque also enables the bacteria to become protected from anti-bacterial agents and environmental stressors [7,8]. The high prevalence of dental caries is a global health problem which reduces the quality of life for those affected. This necessitates the development of an effective therapy to combat this disease [4,9].

The adhesion of *S. mutans* and *S. sobrinus* to the tooth surface is mediated by surface adhesins such as P1 (also known as antigen I/II or PAc), which interact with salivary proteins [10,11], and exopolysaccharides (EPSs) produced by bacteria-associated enzymes such as glucosyltransferases (Gtfs) and, to a lesser extent, fructosyltransferase (Ftf) [12,13,14]. The Gtfs promote the production of glucose polymers called glucans which are predominantly made of α-1.3 (water-insoluble) and α-1.6 (water-soluble) glycosidic linkages [12]. Ftf catalyzes the production of fructans which are β-2,1- and β-2,6-linked homopolymers of fructose [15]. The EPS produced by *S. mutans* binds to the bacterial surface and supports the adherence of the bacteria to the tooth surface [13]. The microbiota of the oral cavity is dynamic and significantly influenced by carbohydrate intake [3,15]. The expression and activities of Gtfs and Ftf are influenced by the sugar source, among them sucrose increasing their expression [16,17]. This response contributes to the caries-promoting effect of sucrose [18].

Many approaches are used to reduce the burden of cariogenic bacteria in the oral cavity with the aim of improving oral health [19,20,21]. These include mechanical removal by tooth brushing and flossing, removal of dental plaques by dentists, and the use of toothpastes and mouthwashes containing fluoride, calcium phosphate, zinc ions, detergents such as sodium lauryl sulfate and antiseptic compounds such as chlorhexidine (CHX), cetylpyridinium chloride and triclosan [19]. Although these approaches have led to an improvement in oral health over the years, there is still a need to develop new formulations against tooth decay.

The polyunsaturated 20:4 (ω-6) fatty acid arachidonic acid (AA) has been shown to exert anti-bacterial and anti-biofilm activities against various Gram-positive bacteria including *S. mutans*, *Streptococcus pneumoniae* and *Staphylococcus aureus*, at concentrations which are non-toxic to normal epithelial cells [22,23,24,25,26,27]. The anti-bacterial activity of AA against the Gram-positive bacteria was related to membrane hyperpolarization, anti-oxidant activity, lipid peroxidation and inhibition of fatty acid synthesis [22,25,26]. AA was shown to reduce the virulence of enterohemorrhagic *Escherichia coli* (EHEC) by being converted by the acyl-CoA synthetase FadD into arachidonic acid-CoA, which binds to FadR and prevents the transcription of its target genes [28]. Supplementation of AA to mice affected the composition of the gut microbiota [29]. AA has also been used in the treatment of *Schistosoma* infections in children with good outcome and safety [30,31,32]. AA is an integral component of eukaryote cell membranes where it is usually esterified in the form of phospholipids and affects membrane fluidity [33]. AA is released from the phospholipids by phospholipase A2 (PLA2) and metabolized into bioactive mediators responsible for resolving inflammation and wound healing [33,34,35]. Eggs and animal-based food are diet sources rich in AA [36]. This suggests that AA can be a potential safe drug for dental caries prevention.

**Figure 1:**
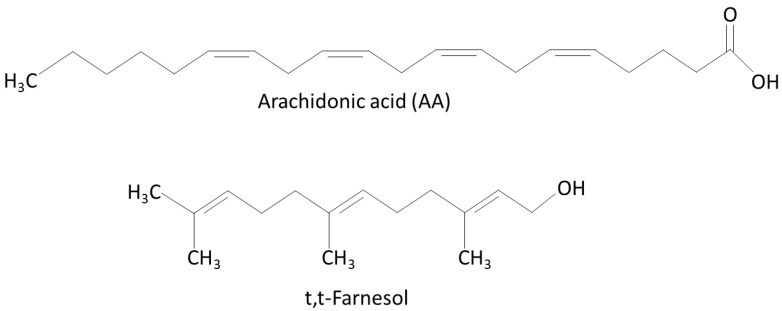
The chemical structures of arachidonic acid (AA) and trans, trans-farnesol (t,t-farnesol).

The current concept of using a combination of anti-microbials with different action mechanisms to reduce bacterial burden has initiated our search for a compound that could enhance the anti-bacterial activity of AA. One molecule that attracted our attention was the sesquiterpene alcohol trans, trans-farnesol (t,t-farnesol). It is a quorum-sensing molecule produced by *Candida albicans*, but it is also present in various plant products such as pine, chamomile, propolis and citrus fruits [37,38]. Besides hindering the ability of *C. albicans* to form hyphae [39,40] and biofilms [41], it has anti-bacterial properties [42,43]. Among others, it affects the growth and biofilm formation of *S. mutans* [44,45] and could prevent enamel demineralization in an in vitro model [46]. t,t-Farnesol acts by reducing glycolytic activity, acid tolerance, virulence-associated gene expression and EPS synthesis by *S. mutans* [44,47]. Both AA and t,t-farnesol have an unsaturated fatty acid chain (Figure 1). Whereas AA has four carbon–carbon double bonds in cis with a carboxyl group at one end, t,t-farnesol has three unsaturated carbon–carbon bonds, two of them being in trans, and a hydroxyl group at the end (Figure 1). Based on their different action mechanisms, we hypothesized that the combination of t,t-farnesol with AA would increase the anti-bacterial activity. The aim of our research was to investigate this hypothesis. Such a combination of natural compounds has not yet been studied against cariogenic bacteria. Investigating their combined effect is innovative as it could potentially be a new anti-bacterial protocol. Here we present data demonstrating that t,t-farnesol indeed increases the anti-bacterial and anti-biofilm effect of AA against *S. mutans* and *S. sobrinus*.

## 2. Results

### 2.1. t,t-Farnesol Enhances the Anti-Bacterial Activity of Arachidonic Acid Against S. mutans and S. sobrinus

**Figure 2:**
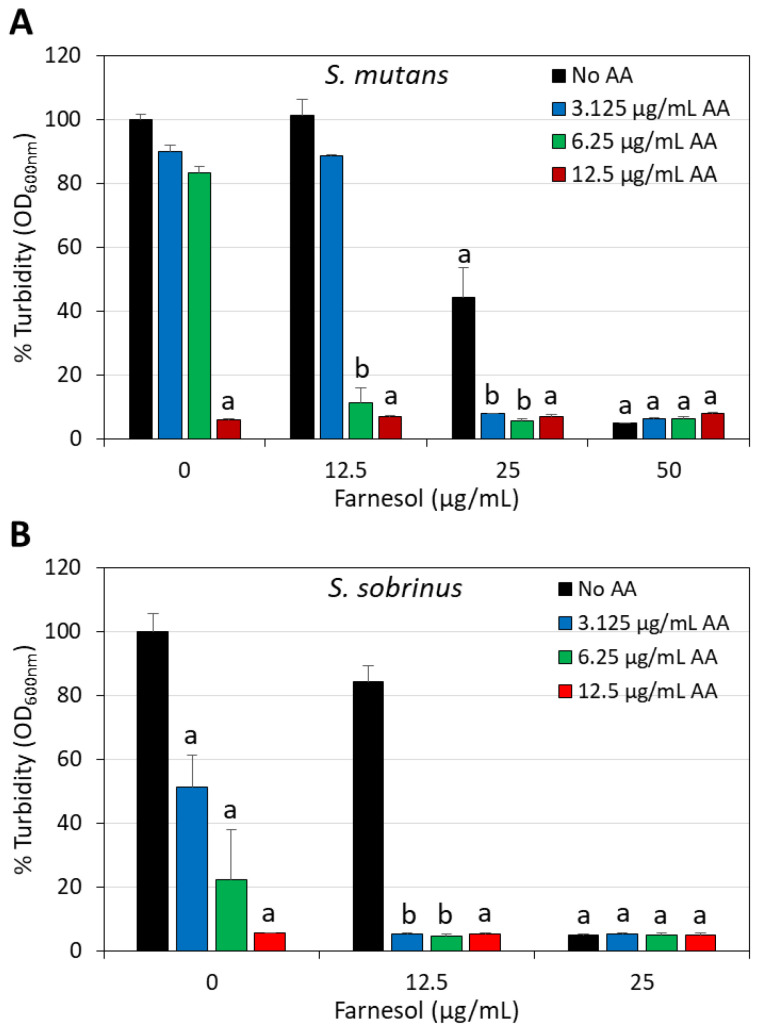
Increased anti-bacterial effect of arachidonic acid and t,t-farnesol against *S. mutans* (**A**) and *S. sobrinus* (**B**) when combined together. The graphs present the percentage turbidity of planktonic growing bacteria after a 24 h incubation with different concentrations of AA and farnesol. N = 3. a: *p* < 0.05 when compared to control bacteria. b: *p* < 0.05 when compared to single treatments.

Since both arachidonic acid (AA) and t,t-farnesol have been documented to have anti-bacterial activity against cariogenic bacteria [22,44,45], we decided to investigate whether these two compounds could enhance the anti-bacterial effect when combined together. To this end, we performed a checkerboard assay of increasing dosages of each compound alone or in combination (3.125–12.5 µg/mL for AA, and 1.56–25 µg/mL for t,t-farnesol) and analyzed the viability of *S. mutans* and *S. sobrinus* after a 24 h incubation by measuring the turbidity. The MIC of AA was 12.5 µg/mL for both bacteria, while the MIC of t,t-farnesol was 50 µg/mL for *S. mutans* and 25 µg/mL for *S. sobrinus* (Figure 2). Although the MIC of AA was the same for both bacteria, *S. sobrinus* showed higher susceptibility to AA than *S. mutans*, as seen by a 78 ± 16% reduction in turbidity at 6.25 µg/mL AA and a 49 ± 10% reduction at 3.125 µg/mL AA, which is in contrast to *S. mutans* with a less than 20% reduction at these concentrations (Figure 2). When AA was combined with sub-MICs of t,t-farnesol, the MIC of AA was reduced 2–4-fold (Figure 2), providing a fractional inhibitory concentration index )FICI) of 0.75. This suggests that the augmented anti-bacterial activity of the combined treatment is an additive effect.

### 2.2. Combined Arachidonic Acid (AA)/t,t-Farnesol Treatment Increased Membrane Perforation

**Figure 3:**
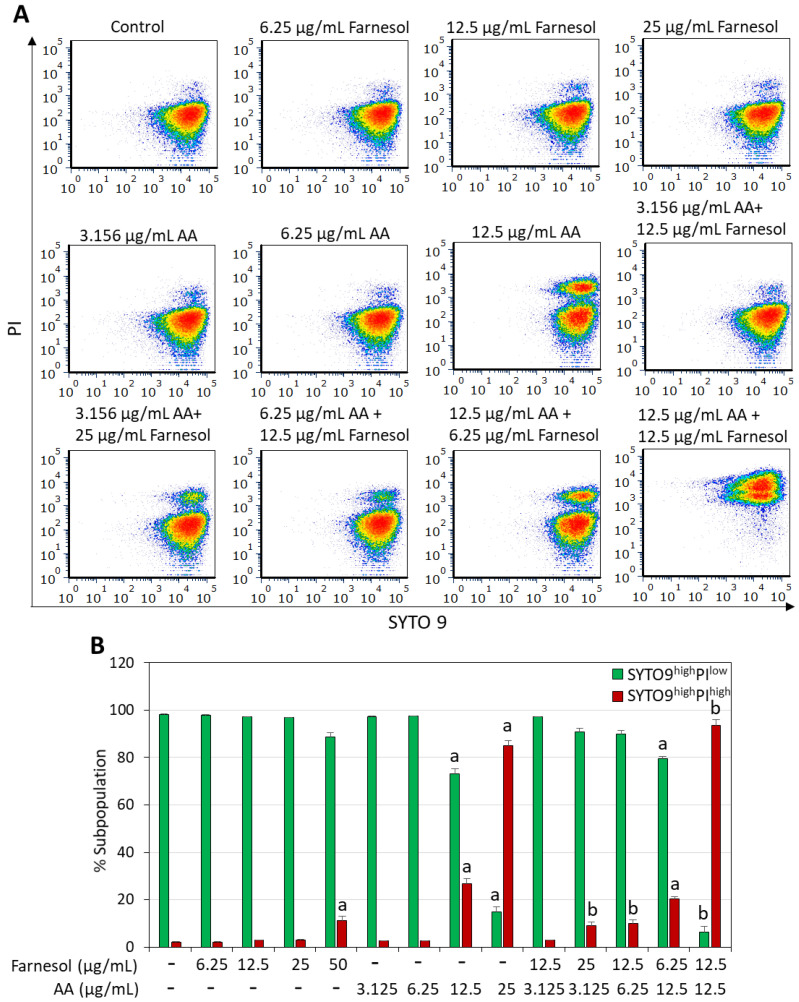
Membrane perforation caused by arachidonic acid (AA) is aggravated when *S. mutans* is co-treated with t,t-farnesol. (**A**). Density dot plots of PI versus SYTO 9 fluorescence intensities of *S. mutans* that was treated with the indicated compounds for 2 h. The samples were analyzed by flow cytometry. The different colors represent the density of the events at a given position. (**B**). Percentage of live (SYTO 9^high^PI^low^) and dead (SYTO 9^high^PI^high^) bacteria, respectively. N = 3. a: *p* < 0.05 when compared to control bacteria. b: *p* < 0.05 when compared to single treatments.

**Figure 4:**
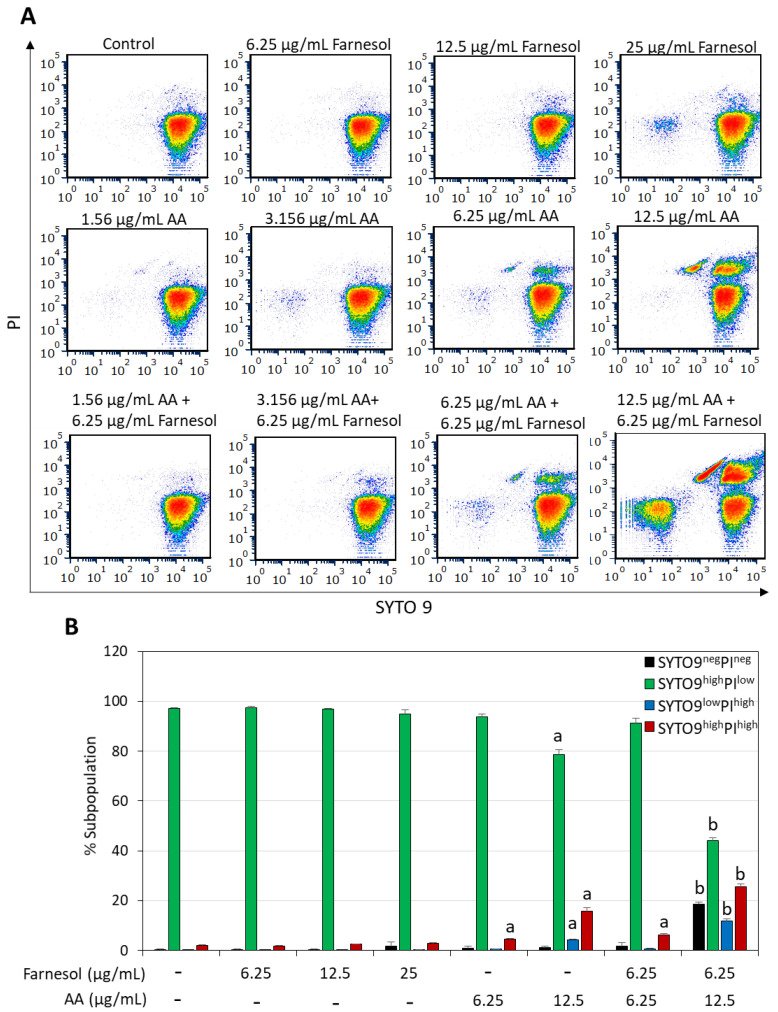
Increased membrane perforation when *S. sobrinus* is co-treated with arachidonic acid (AA) and t,t-farnesol. (**A**). Density dot plots of PI versus SYTO 9 fluorescence intensities of *S. sobrinus* that was treated with the indicated compounds for 2 h. The samples were analyzed by flow cytometry. The different colors represent the density of the events at a given position. (**B**). Percentages of live (SYTO 9^high^PI^low^) and dead (SYTO 9^high^PI^high^; SYTO 9^low^PI^high^; SYTO 9^neg^PI^neg^) bacteria, respectively. SYTO 9^high^PI^high^, SYTO 9^low^PI^high^ and SYTO 9^neg^PI^neg^ represent three different stages of bacterial cell death: SYTO 9^high^PI^high^ represents bacteria with membrane perforation; SYTO 9^low^PI^high^ represents bacteria with membrane perforation with initial cytoplasmic leakage; while SYTO 9^neg^PI^neg^ represents dead bacteria, which have lost nucleic acids (neg = negative). N = 3. a: *p* < 0.05 when compared to control bacteria. b: *p* < 0.05 when compared to single treatments.

The short-term effect of the combined AA/t,t-farnesol treatment on bacterial viability was determined by exposing the bacteria to different concentrations of these compounds for 2 h, followed by SYTO 9/propidium iodide (PI) live/dead staining and analysis of the fluorescence intensities by flow cytometry. This assay enables the determination of the percentages of dead bacteria within a bacterial population, which is represented by the PI^+^ bacteria and SYTO 9/PI double negative cells (Figure 3 and Figure 4). SYTO 9 is a neutral molecule that can penetrate both live and dead bacteria and emits green fluorescence when bound to nucleic acids, while PI is positively charged, can only penetrate bacteria with perforated membranes and emits red fluorescence when bound to nucleic acids [48]. Within the 2 h timeframe, t,t-farnesol caused only a small increase in PI staining of *S. mutans* even at the high MIC of 50 µg/mL (11.2 ± 1.7% PI^+^ cells versus 1.9 ± 0.25% for control; Figure 3). Also, low percentages of PI^+^ cells were observed in *S. sobrinus* when treated with its MIC (25 µg/mL) of t,t-farnesol for 2 h (3.2 ± 0.2% PI^+^ cells versus 2.3 ± 0.2% for control; Figure 4). AA induced a dose-dependent increase in PI^+^ cells in *S. mutans* (26.8 ± 2.1% and 85.1 ± 1.9% at 12.5 and 25 µg/mL AA, respectively; Figure 3). Similarly, 12.5 µg/mL AA caused the appearance of 20.0 ± 1.7% PI^+^ cells in *S. sobrinus* after a 2 h incubation (Figure 4). The percentage of PI^+^ cells increased to 93.7 ± 2.3% when *S. mutans* was treated with 12.5 µg/mL AA and 12.5 µg/mL t,t-farnesol (Figure 3) and 37.3 ± 2.1% when *S. sobrinus* was treated with 12.5 µg/mL AA and 6.25 µg/mL t,t-farnesol (Figure 4). Interestingly, a third population was observed in *S. sobrinus* with the latter AA/t,t-farnesol concentrations, which appeared negative for both SYTO 9 and PI (18.5 ± 1.0%; Figure 4). The SYTO 9^neg^PI^neg^ population, which co-localized with unstained bacteria, likely represents dead bacteria lacking nucleic acid. This population and the PI^+^-positive population constitute 55.9 ± 3.1% of the entire bacterial culture and represent different stages of dead bacteria (Figure 4). There is also a subpopulation that is still PI^+^ but with lower SYTO 9 fluorescence intensity (SYTO 9^low^PI^high^), which may represent dead bacteria with initial cytoplasmic leakage of SYTO 9. Altogether, these data show that AA causes membrane perforation which is aggravated when the bacteria are co-treated with t,t-farnesol.

### 2.3. Arachidonic Acid (AA) Induced Immediate Membrane Hyperpolarization That Was Increased by t,t-Farnesol

**Figure 5:**
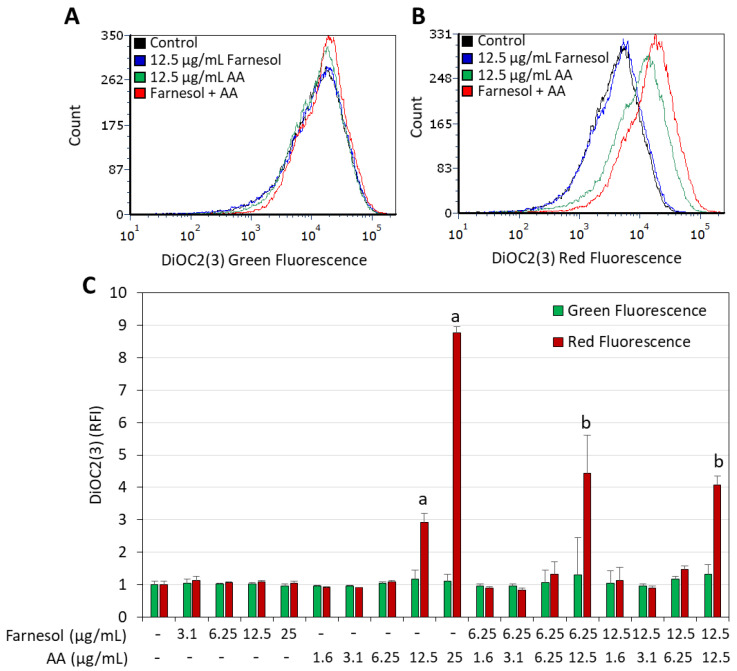
Membrane hyperpolarization induced by arachidonic acid (AA) is intensified when *S. sobrinus* is co-treated with t,t-farnesol. (**A**,**B**). Histograms of DiOC2(3) green fluorescence (**A**) and red fluorescence (**B**) of bacteria exposed to 12.5 µg/mL t,t-Farnesol and/or 12.5 µg/mL AA for 15 min. (**C**). Summary of the relative DiOC2(3) green and red fluorescence intensities of *S. sobrinus* exposed to the indicated concentrations of the two compounds. RFI = Relative fluorescence intensities calculated according to the geometric mean on the De Novo FCS Express 7.12.0007 software. DiOC2(3) is a potentiometric drug whose red fluorescence is intensified upon membrane hyperpolarization, while the green fluorescence intensity is unaffected by the ΔΨ [50,51]. Thus, an increase in the red fluorescence relative to the green fluorescence is an indication of membrane hyperpolarization. N = 3. a: *p* < 0.05 when compared to control bacteria. b: *p* < 0.05 when compared to single treatments.

AA has previously been shown to induce membrane hyperpolarization in *S. mutans* in a dose-dependent manner [22,49]. This led us to study the effect of combined AA/t,t-farnesol treatment on the membrane potential of *S. sobrinus*. This was studied by incubating bacteria that were exposed to the compounds for 15 min with the potentiometric dye DiOC2(3), followed by flow cytometric analysis of green versus red fluorescence intensities. AA, but not t,t-farnesol, as a single compound induced membrane hyperpolarization in *S. sobrinus* in a dose-dependent manner (Figure 5). When the bacteria were treated with both compounds, there was a further increase in membrane hyperpolarization (Figure 5).

### 2.4. Enhanced Anti-Biofilm Activity of Combined Arachidonic Acid (AA)/t,t-Farnesol Treatment Against S. mutans and S. sobrinus

**Figure 6:**
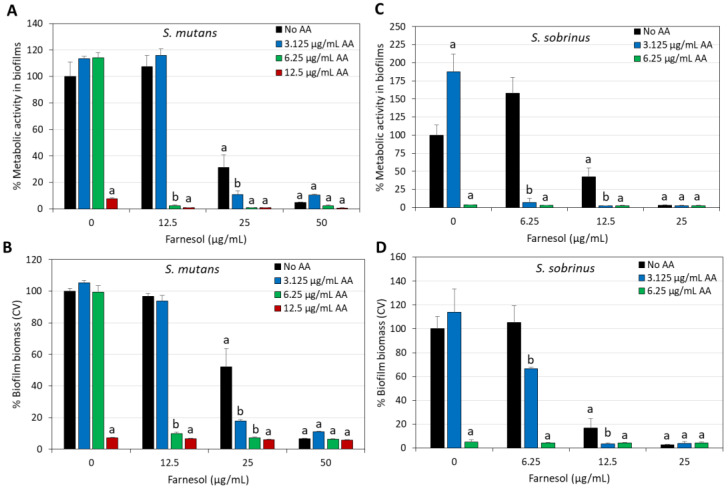
Increased anti-biofilm effects against *S. mutans* (**A**,**B**) and *S. sobrinus* (**C**,**D**) when arachidonic acid (AA) is combined with t,t-farnesol. (**A**,**C**) The metabolic activity of the biofilms after a 24 h incubation with different concentrations of AA and t,t-farnesol, as measured by the MTT assay. (**B**,**D**) The biofilm biomass after a 24 h incubation with different concentrations of AA and t,t-farnesol, as measured by crystal violet (CV) staining. N = 3. a: *p* < 0.05 when compared to control bacteria. b: *p* < 0.05 when compared to single treatments.

**Figure 7:**
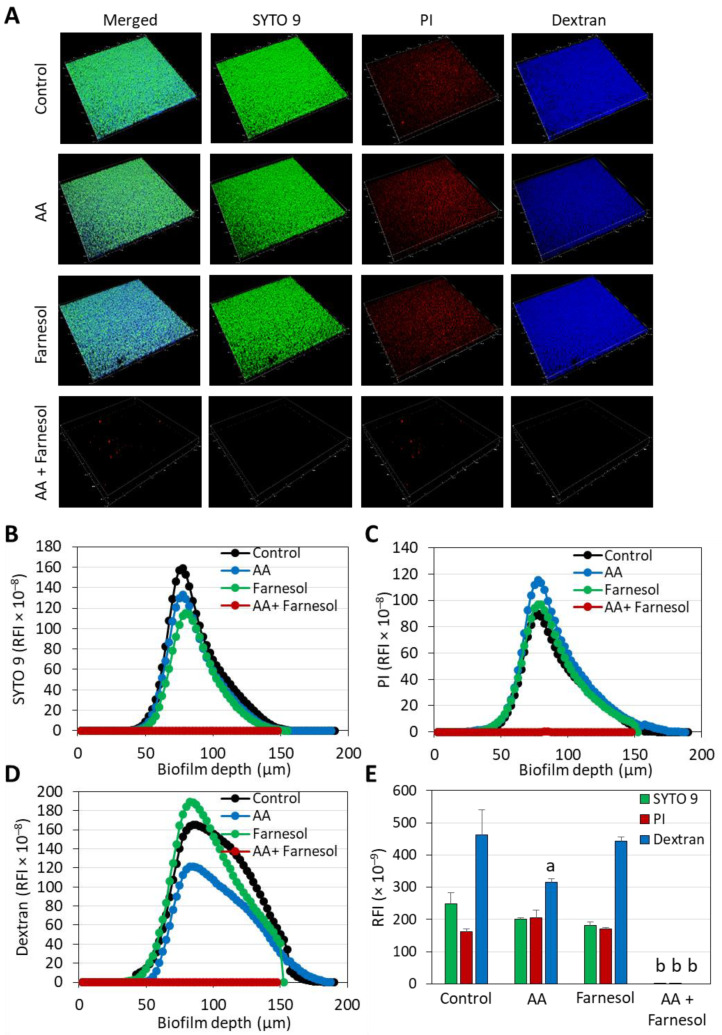
Spinning disk confocal microscopy (SDCM) of *S. mutans* biofilms formed in the absence or presence of 3.125 µg/mL AA and 12.5 µg/mL t,t-farnesol for 24 h. (**A**). The individual and merged fluorescence images of the three stains: SYTO 9 that enters both live and dead bacteria and emits green fluorescence; PI that can only enter bacteria with damaged membrane and emits red fluorescence; and AlexaFluor^647^ Dextran 10,000 that shows the EPS, emits far-red fluorescence and is presented here in blue. The images cover an area of 1497.6 µm × 1497.6 µm. (**B**–**D**) The relative fluorescence intensities (RFI) of SYTO 9 (**B**), PI (**C**) and Dextran 10,000 (**D**) in each of the biofilm layers. The numbers on the Y-axis represent the RFI values calculated by NIS element × 10^−8^. (**E**) The area under the curve (AUC) of the graphs in B-D. Here the total RFI values were multiplied by 10^−9^. N = 3–4. a: *p* < 0.05 when compared to control bacteria. b: *p* < 0.05 when compared to single treatments.

**Figure 8:**
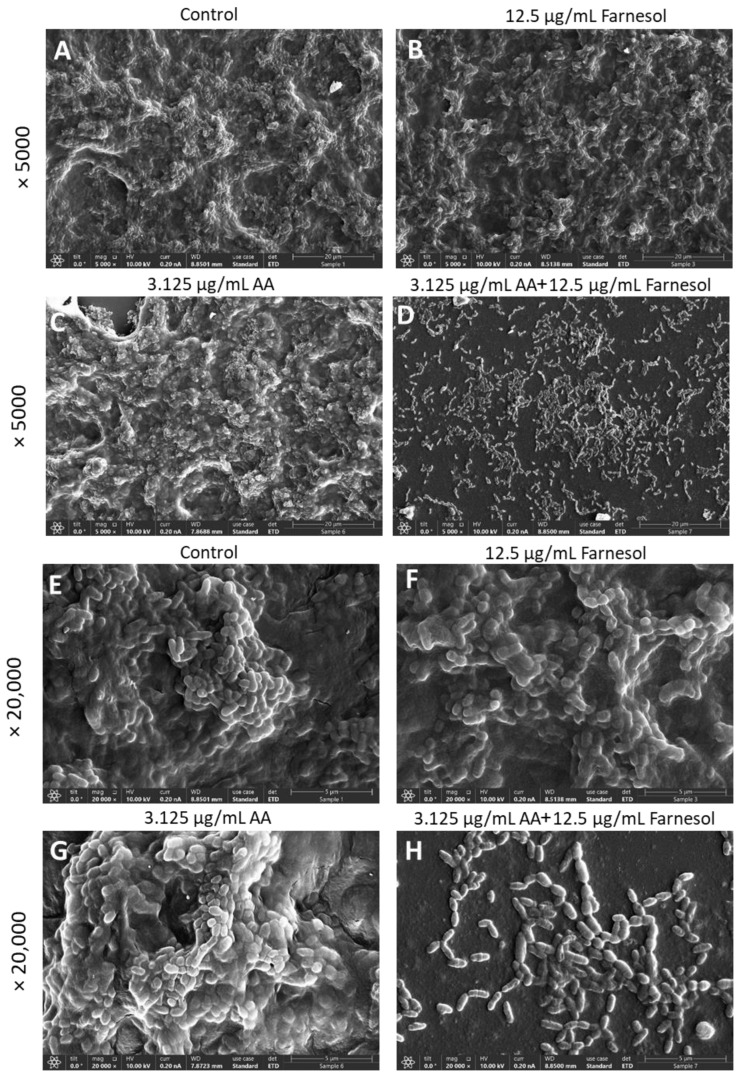
HR-SEM images of *S. mutans* biofilms formed in the absence or presence of 3.125 µg/mL AA and 12.5 µg/mL t,t-farnesol for 24 h. (**A**–**D**) ×5000 magnifications. The bar represents 20 µm. (**E**–**H**) ×20,000 magnifications. The bar represents 5 µm.

It was important not only to study the anti-bacterial activity of the compounds but also to investigate their effects on biofilm formation as well as on preformed, mature biofilms. This is in light of the important role of cariogenic bacterial biofilms on tooth surfaces in tooth decay [52]. Similar to the anti-bacterial effect, there was an additive anti-biofilm effect when AA was combined with t,t-Farnesol (Figure 6). Both the metabolic activity and the total biofilm biomass were reduced (Figure 6). Confocal microscopy and high-resolution scanning electron microscopy (HR-SEM) confirmed the reduced biofilm formation caused by the combined treatment of sub-MBIC concentrations of AA (3.125 µg/mL) and t,t-farnesol (12.5 µg/mL) after a 24 h incubation (Figure 7 and Figure 8). The confocal microscopy was performed on *S. mutans* biofilm samples that were stained with SYTO 9, PI and fluorescent dextran 10,000 (Figure 7). Biofilms of control and singly treated samples showed complete bacterial coverage of the surface (Figure 7A), although 3.125 µg/mL AA and 12.5 µg/mL t,t-farnesol caused a 18.9 ± 2.0% and 27.1 ± 4.7% reduction in SYTO 9 staining, respectively (Figure 7E). Additionally, 3.125 µg/mL AA significantly reduced the EPS by 31.9 ± 2.6% (Figure 7E). On the contrary, there were almost no biofilms (less than 0.0004%) in samples exposed to the combined treatment (Figure 7). The HR-SEM images show multilayered complex biofilms of *S. mutans* in the control and single-treatment samples with EPS, while those exposed to both compounds showed scattered bacteria in a single layer, some of them with distorted structures (Figure 8).

**Figure 9:**
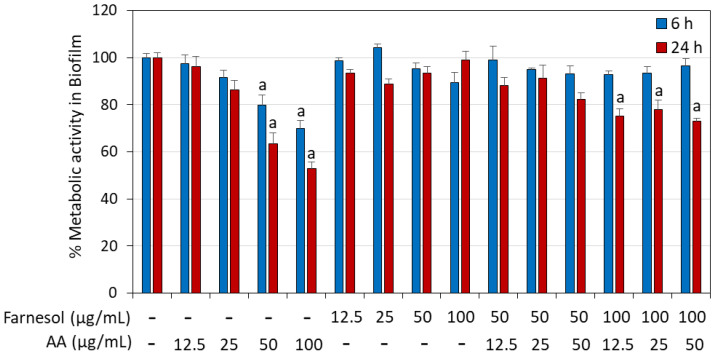
Effect of arachidonic acid (AA) and t,t-farnesol on preformed *S. mutans* biofilms. *S. mutans* was allowed to form biofilms in BHI supplemented with 1% sucrose for 24 h. The mature biofilms were then washed in PBS and exposed to different concentrations of AA and/or t,t-farnesol for 24 h, and the metabolic activity of the remaining biofilms was measured using the MTT assay. N = 3. a: *p* < 0.05 when compared to control biofilms.

It was also important to investigate whether AA and/or t,t-farnesol could act on preformed, mature biofilms. To this end, *S. mutans* biofilms were allowed to form for 24 h prior to exposure to the compounds for 6 and 24 h. Only AA at 50 µg/mL and 100 µg/mL reduced the metabolic activity of the biofilms, reaching up to a 47.2 ± 2.8% reduction after a 24 h incubation (Figure 9). t,t-Farnesol did not enhance the effect of AA on preformed biofilms (Figure 9).

### 2.5. Arachidonic Acid and t,t-Farnesol Reduced the Expression of Some Biofilm-Related Genes

**Figure 10:**
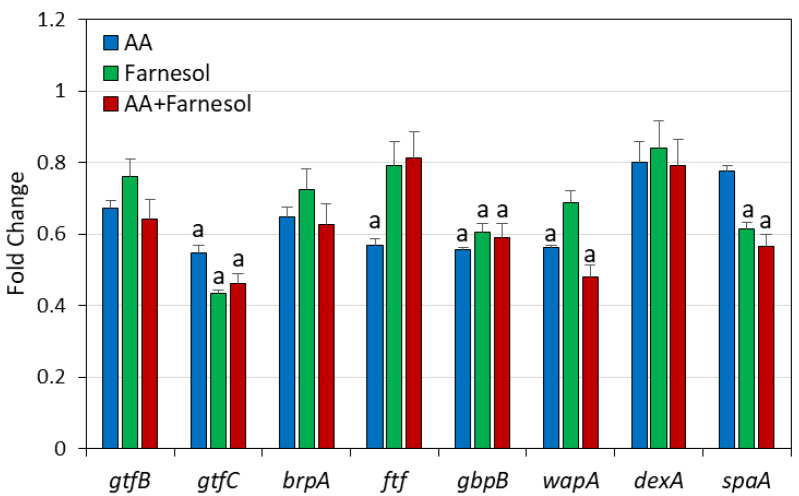
Effect of arachidonic acid (AA) and t,t-farnesol on the expression of biofilm-related genes. *S. mutans* was exposed to 3.125 µg/mL AA and/or 12.5 µg/mL t,t-farnesol for 2 h prior to RNA isolation. The gene expression was determined by semiquantitative real-time PCR using *gltA* and *glnA* as internal standards, and the relative expression was calculated against control bacteria that were set to 1. N = 3. a: *p* < 0.05 when compared to control bacteria.

The effect of the compounds on the expression of genes involved in biofilm formation was studied after exposing *S. mutans* to 3.125 µg/mL AA and/or 12.5 µg/mL t,t-farnesol for 2 h. The short incubation time was chosen in order to detect early effects of the compounds on gene expression. AA and t,t-farnesol alone reduced the expression of some of the biofilm-related genes, among them *gtfC* and *gbpB* were the most affected with a 40–50% reduction (Figure 10). *wapA* was significantly reduced by AA, while *spaA* was significantly reduced by t,t-farnesol (Figure 10). However, there was no further reduction in the expression of these genes by combined treatment.

## 3. Discussion

A balanced oral microbiome is critical for maintaining oral health, but frequent consumption of dietary carbohydrates can disrupt this homeostasis and lead to dysbiosis [53,54]. Dietary carbohydrates are fermented to lactic acid by cariogenic bacteria such as *S. mutans* and *S. sobrinus*, which lowers the pH in their local microenvironment [1]. Since these bacteria are able to survive under acidic conditions, they will relatively take over the oral bacterial population at the expense of other oral microorganisms [1]. The dietary carbohydrates also induce biofilm-related genes in these bacteria that increase their adherence to abiotic and biotic surfaces, where biofilm formation on the tooth surface, the dental plaque, is a critical step for tooth decay [55].

Understanding the caries-associated microbiome is crucial for developing strategies to reverse dysbiosis and achieve desirable oral health [56]. Recent studies have searched for natural compounds that may have beneficial health properties, among them the polyunsaturated 20:4(ω-6) arachidonic acid (AA), found in various food sources (e.g., eggs, fish and poultry), has attracted attention [33,35]. Since AA has anti-bacterial activities against several Gram-positive bacteria, including the cariogenic *S. mutans* [22,23,24,25,26], it might have potential uses in the control of dental caries. In the present study, AA was also shown to be active against *S. sobrinus*. We were interested in finding compounds that could increase the anti-bacterial activity of AA with the aim of developing a composite formulation with higher anti-caries efficacy. In a previous study, the two antiseptics commonly used in dentifrices, chlorhexidine and cetylpyridinium chloride, were found to antagonize the anti-bacterial activity of AA, while triclosan had an additive effect [49]. As the current trend is to reduce the use of these antiseptics due to undesired side effects and the development of drug resistance [57,58], we searched for a natural compound that could fulfill the goal.

Here, we document that trans, trans-farnesol (t,t-farnesol), a quorum-sensing mediator of fungi and also commonly found in propolis and citrus fruits [37,38], enhances the anti-bacterial and anti-biofilm activity of AA against *S. mutans* and *S. sobrinus*. The combination of sub-minimum inhibitory concentration (MIC) levels of these two compounds significantly reduced bacterial viability and prevented biofilm formation. The anti-bacterial activity of the dual treatment was demonstrated by microtiter plate assay (Figure 2) and by an early increase in propidium iodide uptake indicative of membrane perforation (Figure 3 and Figure 4). AA caused membrane hyperpolarization which was enhanced by t,t-farnesol, although the latter did not itself induce membrane hyperpolarization (Figure 5). Altered membrane polarization is a sign of an imbalance in membrane ion transport, which may have implications for bacterial metabolism and their ability to survive under acidic conditions [59]. We showed that membrane hyperpolarization preceded propidium iodide uptake (15 min versus 2 h, respectively; Figure 5 versus Figure 3 and Figure 4), which is an indication for bacterial cell death. The implication of this finding is that the dual treatment is bactericidal for the two cariogenic bacteria tested and is thus expected to be able to reduce bacterial burden in the oral cavity. The dual treatment allows the use of lower concentrations of each compound, which is expected to reduce the appearance of possible adverse effects. Another advantage is that both compounds are naturally occurring, found in common food ingredients, and therefore are expected to have low cytotoxicity. t,t-Farnesol has previously been shown to have anti-bacterial activities against oral *Streptococci* [44,45,46], and the researchers proposed t,t-farnesol as a potential drug for improving oral health.

The action mechanisms of AA and t,t-farnesol appear to differ and thus complement each other. AA induces membrane hyperpolarization ([22] and Figure 5) and membrane perforation ([22] and Figure 3 and Figure 4), alters transmembrane transport [22], induces lipid peroxidation with the production of cytotoxic free radicals [25] and impairs fatty acid synthesis by inhibiting enoyl-acyl carrier protein reductase (FabI) and downregulating genes involved in fatty acid biosynthesis [26,60]. t,t-Farnesol has been shown to increase membrane proton permeability [44], reduce glycolytic activity [44] and decrease EPS production [61] in *S. mutans*. It is worth mentioning that AA may also have an indirect anti-bacterial effect by increasing NADPH-dependent superoxide production in macrophages and neutrophils, leading to enhanced killing of engulfed microorganisms [24]. t,t-Farnesol also suppresses biofilm formation by *C. albicans* monocultures and mixed *C. albicans*/*S. mutans* co-cultures [41]. The dual anti-microbial function of t,t-farnesol is beneficial as the presence of *C. albicans* together with *S. mutans* in dental plaque is associated with worsening caries conditions in children [62,63].

Another important feature of AA and t,t-farnesol is their ability to reduce biofilm formation of *S. mutans* and *S. sobrinus* as single agents and to further reduce biofilm formation when used together (Figure 6). The anti-biofilm activity of the combined treatment as observed by crystal violet biomass staining and MTT metabolic assay goes along with the anti-bacterial activity under planktonic conditions, implying that the anti-biofilm effect is partly due to the anti-bacterial activity (Figure 6 versus Figure 1). However, we cannot exclude the possibility that the compounds also have direct anti-biofilm activity. Gene expression studies show a reduction in the expression of some biofilm-related genes, especially *gtfC*, *gbpB*, *wapA* and *spaA* (Figure 10), which may contribute to reduced biofilm formation (Figure 6). GtfC is a glycosyltransferase involved in the synthesis of both water-soluble and water-insoluble glucans that make up the biofilm matrix and promote bacterial adhesion to surfaces [64]. GbpB is a glucan-binding protein involved in sucrose-dependent biofilm formation [65]. WapA can interact with collagen [66] but is also an amyloidogenic protein which forms part of the fibrillar extracellular matrix of biofilms [67]. SpaA (also termed SpaP or I/II antigen) [68] is a surface-associated adhesin involved in saliva-mediated aggregation and attachment to the tooth surface [7]. Thus, the downregulation of these genes by AA/t,t-farnesol may contribute to the reduced biofilm formation. Previous studies by Koo et al. have shown that t,t-farnesol could act together with apigenin to reduce biofilm formation by *S. mutans* [61], and the anti-caries effect could be further enhanced by the simultaneous addition of sodium fluoride [69]. Fluoride has also been shown to increase the anti-bacterial and anti-biofilm activity of AA [49]. The monounsaturated fatty acid oleic acid was shown to directly inhibit glycosyltransferase activity [70]. It remains to be tested whether this also applies to AA.

Confocal microscopy and HR-SEM imaging confirmed the anti-biofilm activity of the dual treatment of AA with t,t-farnesol, where only residual bacteria could be detected attached to the surface (Figure 7 and Figure 8). AA, but not farnesol, also had anti-biofilm activity on mature, preformed biofilms (Figure 9). It is well known that it is much more difficult to eliminate preformed biofilms than to prevent biofilm formation, which is due to lower penetration of the drug through the biofilm matrix, the sessile state of the bacteria in the biofilms and the higher robustness of the biofilm-associated bacteria, among others [55]. Based on the data from this research, we can conclude that dual treatment with AA and t,t-farnesol is likely to be more effective in preventing plaque formation than in reducing existing plaques. The limitations of this study are that it is only an in vitro study, and the effect of the combined treatment was only tested against two cariogenic bacterial strains. To prove the applicability of our approach, in vivo studies with mouthwashes or toothpaste containing AA and t,t-farnesol should be performed using caries tooth models at the preclinical stage, and thereafter clinical trials should be conducted to test whether such treatment can reduce the prevalence of dental caries.

## 4. Materials and Methods

### 4.1. Materials

Arachidonic acid (AA) (>99% purity; Nu-Check Prep, Elysian, MN, USA) was dissolved in ethanol (HPLC-grade, Baker, Gliwice, Poland) to a final concentration of 50 mg/mL and stored at −20 °C. Trans, trans-farnesol (t,t-farnesol; Sigma, St. Louis, MO, USA) was dissolved in ethanol to a final concentration of 20 mg/mL. The various assays described below were performed with each compound alone or in combination in a checkerboard assay in the concentration range of 1.25–100 μg/mL, with the exception of SDCM, HR-SEM and gene expression analysis, where selected concentrations were used as indicated in the figures. Equal ethanol concentrations (0.005–0.7%) were included in the experiments as controls. All assays were performed at least in triplicates.

### 4.2. Bacteria and Cultivation Conditions

*Streptococcus mutans* UA159 (ATCC 700610) and *Streptococcus sobrinus* (ATCC 27351) were cultivated in brain–heart infusion (BHI) broth (HiMedia Laboratories Pvt. Ltd., Maharashtra, India) for planktonic growth and in BHI supplemented with 2% sucrose (BHIS) for biofilm formation [71]. The day before the experiment, 100 µL of a frozen bacterial stock (−80 °C) was inoculated in 10 mL BHI and incubated overnight at 37 °C in a humidified incubator in the presence of 5% CO_2_. The morphology of the bacteria was verified under a light microscope (Axio Lab.A1, Carl Zeiss GmbH, Jena, Germany) at a ×1000 magnification showing ovoid bacteria in chains. The purity of the bacterial cultures was verified by seeding the bacteria on BHI-agar plates, with the appearance of tiny colonies characteristic of these bacteria.

### 4.3. Microplate Viability Assay of Planktonic Growing Bacteria

To measure the effect of the compounds on planktonic bacterial growth, a checkerboard assay was performed [49]. Overnight bacterial cultures were diluted to an optical density (OD_600nm_) of 0.1 and incubated for 24 h at 37 °C with different concentrations and combinations of the two compounds, AA (1.25–100 μg/mL) and t,t-farnesol (1.25–100 μg/mL), in 200 μL BHI per well of 96-well flat-bottom tissue culture grade plates (Corning Incorporation, Kennebunk, ME, USA) [22]. At the end of incubation, the OD at 600 nm of the bacterial cultures was measured in a Multiskan SkyHigh microplate reader (ThermoScientific, Life Technologies Holdings Pte Ltd., Singapore). Equal ethanol concentrations (reaching a maximum of 0.7%) served as controls. The percentage viability was measured by the following formula: (OD_sample_ − OD_background_)/(OD_control_ − OD_background_) × 100%. The minimum inhibitory concentration (MIC) was defined as the lowest concentration resulting in no visible bacterial growth after a 24 h incubation. No visible growth was defined as the OD_600nm_ of treated samples reaching the OD_600nm_ of blank samples. To determine whether the combined treatment is synergistic, additive or antagonistic, the fractional inhibitory concentration index (FICI) value [72] was determined by the following formula: FICI = (MIC_A_ in combination/MIC_A_ alone) + (MIC_F_ in combination/MIC_F_ alone) where A is arachidonic acid and F farnesol.

### 4.4. Biofilm Assays

#### 4.4.1. Effect of Single and Combined Treatments on Biofilm Formation

To test the effect of the combined treatment on biofilm formation, the bacteria were diluted to an initial OD_600nm_ of 0.1 in BHI broth supplemented with 2% sucrose (BHIS) to induce biofilm formation conditions [22]. The bacteria were incubated in the absence or presence of various concentrations and combinations of the two test compounds, AA (1.25–100 μg/mL) and t,t-farnesol (1.25–100 μg/mL), or equal ethanol concentrations in 200 μL BHI in a checkerboard assay. After a 24 h incubation at 37 °C, the biofilms were washed twice with phosphate buffered saline (PBS) to remove non-adherent cells. The metabolic activity was determined by exposing the biofilms to 50 μL of a 0.5 mg/mL MTT solution in PBS for 1h at 37 °C. The formazan formed by the enzymatic reduction of MTT in the live bacteria was dissolved in 200 μL of dimethylsulfoxide (DMSO), and the absorbance at 570 nm was measured in a Multiskan SkyHigh microplate reader. The biofilm biomass was determined by staining the biofilms with 0.25% crystal violet (CV) in ddw (prepared by diluting 1% Gram crystal violet solution (Merck KGaH, Darmstadt, Germany) in ddw) for 20 min at room temperature. Thereafter, the biofilms were washed with double distilled water (ddw) to remove excess stain, and the absorbance was read at 595nm in a Multiskan SkyHigh microplate reader. The minimum biofilm inhibitory concentration (MBIC) was defined as the lowest concentration of the compounds required for no visible biofilm after a 24h incubation. The relative amount of biofilms was determined by the following formula: (OD_sample_ − OD_background_)/(OD_control_ − OD_background_) × 100%.

#### 4.4.2. Effect of Single and Combined Treatments on Preformed Biofilms

To determine the effect of the compounds on preformed biofilms, the bacteria were allowed to form biofilms in BHIS for 24 h prior to exposure to different combinations of the agents for another 24 h. At the end of incubation, the biofilms were washed with PBS and the metabolic activity determined by the MTT assay as described above in Section 4.4.1.

### 4.5. SYTO 9/Propidium Iodide (PI) Live/Dead Staining by Flow Cytometry

The bacteria with an initial OD_600nm_ of 0.3 were incubated in the absence or presence of the different combinations of the test compounds in 1 mL BHI for 2 h. At the end of incubation, the samples were centrifuged at 5000× *g* for 5 min, and the bacterial pellet was resuspended in 1 mL PBS containing 1 µM SYTO 9 (Molecular Probes, Life Technologies, Carlsbad, CA, USA) and 2 µg/mL propidium iodide (PI) (Sigma, St. Louis, MO, USA) [22]. Following a 20 min incubation at room temperature, the fluorescence intensities were measured in an LSR-Fortezza flow cytometer (BD Biosciences, San Jose, CA, USA) using the excitation/emission of 488 nm/540 nm for SYTO 9 and 561 nm/600 nm for PI. A total of 50,000 events were collected for each sample using BD FACSDiva software 8.0.1, and each treatment group was performed in triplicate. The De Novo FCS Express 7.12.0007 software was used for analyzing the collected data. SYTO 9 is a neutral nucleic acid dye that can penetrate both live and dead bacteria, while PI is a positively charged nucleic acid dye that can only penetrate the membranes when perforated. Live bacteria are SYTO 9^high^PI^low^, while dead bacteria with perforated membrane can appear in three different subpopulations according to the extent of membrane perforation and cytoplasmic leakage: SYTO 9^high^PI^high^ (membrane perforation), SYTO 9^low^PI^high^ (membrane perforation with cytoplasmic leakage) and SYTO 9^neg^PI^neg^ (loss of nucleic acids).

### 4.6. Determination of Membrane Polarization by Flow Cytometry

Bacteria with an initial OD_600nm_ of 0.3 were centrifuged and the pellets resuspended in 1 mL PBS containing different combinations of the test compounds or ethanol. After a 15 min incubation, the potentiometric dye DiOC2(3) (BacLight Membrane Potential Kit, Molecular Probes, Life Technologies, Eugene, OR, USA) was added to each sample except for blank samples to a final concentration of 30 µM [22]. After 20 min, the green and red fluorescence intensities were determined by flow cytometry using the excitation/emission of 488 nm/530 nm for green fluorescence and 488 nm/620 nm for red fluorescence. The relative fluorescence intensities (RFIs) of green and red fluorescence were calculated according to geometric mean obtained by the De Novo FCS Express 7.12.0007 software, setting control to 1. The geometric mean on flow cytometry is the Nth root of the product of all observed values. A total of 50,000 events were analyzed for each sample in triplicate. A relative increase in the red fluorescence intensity versus green fluorescence intensity is an indication for membrane hyperpolarization.

### 4.7. Scanning Disk Confocal Microscopy (SDCM) Imaging for Determining Live/Dead Bacteria and Biofilm Structure and Depth

Bacteria were allowed to form biofilms in the absence or presence of different combinations of the compounds in 300 µL BHIS per well of ibiTreat 8-well µ-slides (Ibidi GmbH, Gräfelfing, Germany) for 24 h at 37 °C. AlexaFluor^647^-conjugated Dextran 10,000 (Molecular Probes Inc., Eugene, OR, USA) was added to a final concentration of 5 µg/mL during incubation to stain EPS. On the following day, the biofilms were washed twice with 300 µL PBS and stained with 200 µL of 3.3 µM SYTO 9 and 5 µg/mL PI in PBS for 20 min at room temperature. The stained biofilms were washed again twice with 300 µL PBS, fixed in 4% paraformaldehyde (Electron Microscopy Sciences, Hatfield, PA, USA) for 20 min at room temperature and mounted in 200 µL 50% glycerol prior to visualization using a scanning disk confocal microscope (Nikon Corporation, Tokyo, Japan). Images were captured at 2.5 µm intervals from the bottom to the top of the biofilm, using the excitation laser at 488 nm for SYTO 9 (green fluorescence; live bacteria), the excitation laser at 561 nm for PI (red fluorescence; dead bacteria) and the excitation laser at 640 nm for AlexaFluor^647^-conjugated Dextran 10,000 (far-red fluorescence, presented in the images in blue; EPS staining) [71]. NIS element AR (Advanced Research) software version 5.21.03 (Nikon Instruments Inc.) was used to analyze the resulting 3D images and quantify the fluorescence intensities in each layer of each biofilm. The analysis of all images was performed keeping all parameters the same.

### 4.8. High-Resolution Scanning Electron Microscopy (HR-SEM) of Biofilms

To investigate the effect of the combined treatment on the bacterial morphology and biofilm structure, the bacteria were incubated on 0.5 cm × 0.5 cm glass slide pieces under biofilm-forming conditions in the absence or presence of the compounds. After a 24 h incubation, the biofilms formed on the glass pieces were washed with ddw, fixed in 4% glutaraldehyde (Electron Microscopy Sciences, Hatfield, PA, USA) in ddw for 2 h, washed again in ddw and dried on glass pieces. The samples were then coated with iridium and visualized by an analytical high-resolution scanning electron microscope (HR-SEM) (Apreo 2S LoVac, ThermoScientific) at various magnifications [22].

### 4.9. Gene Expression Analysis

To study the effect on gene expression, the bacteria were incubated at an initial OD_600nm_ of 0.1 in 20 mL of BHIS in the absence or presence of test compounds for 2 h, and then RNA was isolated using Tri-reagent as described by Chamlagain et al. [22]. The RNA was reverse transcribed to cDNA using the AB high-capacity cDNA reverse transcription kit (Applied Biosystems, Vilnius, Lithuania), and the relative gene expression was determined by quantitative real-time PCR using 10 ng cDNA per reaction, 300 nM primer mix (Table 1) and Luna Universal qPCR Master Mix (New England BioLabs, Inc., Ipswich, MA, USA) in a Bio-Rad CFX96 Real-Time Detection System (Bio-Rad Laboratories, Inc., Hercules, CA, USA). The relative gene expression was determined by the 2^−ΔΔCt^ method using *gltA* and *glnA* as house-keeping genes and calculated against control bacteria which were set to 1. A reduction by more than 35% was considered significant.

### 4.10. Statistical Analysis

The experiments were performed in triplicates or quadruplicates, and the obtained data were processed using the Microsoft Excel program. Data are presented as the average ± standard deviation from a representative experiment. Statistical significance of the different treatments was determined by Student’s *t*-test and by one-way ANOVA with ad hoc corrections. Differences were considered statistically significant when the *p*-value was less than 0.05. Data obtained from single treatments were compared to control samples and labeled with the letter “a” in the figures when the *p*-value was less than 0.05. Data obtained from combined treatments were compared to each of the single treatments and labeled with the letter “b” in the figures when the *p*-value was less than 0.05.

## 5. Conclusions

The overall aim of this in vitro study was to demonstrate that the combination of arachidonic acid (AA) with t,t-farnesol can achieve an enhanced anti-bacterial and anti-biofilm effect against the cariogenic bacteria *S. mutans* and *S. sobrinus* compared to the single treatments. The use of these two compounds, which have different anti-bacterial mechanisms, lowered the effective dosage of each compound required to limit both bacterial growth and biofilm formation of the tested cariogenic bacteria. The clinical implication of this study is that the combination of AA with t,t-farnesol can be considered a valuable, safe treatment strategy with potential benefits in reducing dental caries. The natural origin of the two compounds supports their use in the clinic to maintain a healthy oral environment. Further in vivo studies are required to prove the applicability of the two compounds in preventing dental caries.

## Figures and Tables

**Table 1:** The primer sequences used for real-time PCR of *Streptococcus mutans*.

Gene Symbol	Forward Primer	Reverse Primer	Reference
*brpA*	GGAGGAGCTGCATCAGGATTC	AACTCCAGCACATCCAGCAAG	[73]
*dexA*	TATTTTAGAGCAGGGCAATCG	AACCTCCAATAGCAGCATAAC	[74]
*ftf*	AAATATGAAGGCGGCTACAACG	CTTCACCAGTCTTAGCATCCTGAA	[22]
*gbpB*	AGGGCAATGTACTTGGGGTG	TTTGGCCACCTTGAACACCT	[22]
*glnA*	CCTTGGGGAGATGAAAACGGAGCCG	TGGCCATAAAGGTTGCATACAAACC	[75]
*gltA*	TGCCTTAACGATGTTAGAGAGAATG	AAAGACTATCTTCAAAAGCACACCC	[75]
*gtfB*	AGCAATGCAGCCAATCTACAAAT	ACGAACTTTGCCGTTATTGTCA	[22]
*gtfC*	GGTTTAACGTCAAAATTAGCTGTATT	CTCAACCAACCGCCACTGTT	[22]
*spaA*	GACTTTGGTAATGGTTATGCATCAA	TTTGTATCAGCCGGATCAAGTG	[22]
*wapA*	GCACGCTTGCAGTACATTGC	CATAAGGTCGCGAGCAGCT	[71]

## Data Availability

Raw data are available upon reasonable request.
